# Trait Self-Control Discriminates Between Youth Football Players Selected and Not Selected for the German Talent Program: A Bayesian Analysis

**DOI:** 10.3389/fpsyg.2019.02203

**Published:** 2019-09-26

**Authors:** Wanja Wolff, Alex Bertrams, Julia Schüler

**Affiliations:** ^1^Institute of Sport Sciences, University of Konstanz, Konstanz, Germany; ^2^Institute of Educational Science, University of Bern, Bern, Switzerland

**Keywords:** personality, football/soccer, talent, Bayes factor, expertise

## Abstract

Trait self-control predicts success in various walks of life. Sports is a prototypical domain, where self-control is required, and there is evidence that successful athletes display superior self-control. Here, we assess if self-control already differs between athletes that were selected for a talent development program and non-selected athletes. Self-reported trait self-control was assessed in *n* = 25 (7 = female, 13.2 ± 1.7 years) youth football players who were part of the German talent development program and in *n* = 27 (6 = female, 13.6 ± 1.8 years) age and sex matched youth football players, who trained at the same clubs but had not been selected for the program. A one-sided Bayesian two-sample *t*-test yielded a Bayes factor of 54.99, indicating very strong evidence for the hypothesis that elite youth football players have higher trait self-control than non-elite youth football players. The 95% credibility interval indicates that the true value of δ lies between 0.28 and 1.42, indicating some uncertainty regarding the effects’ magnitude. We show that already at young age, elite athletes display higher levels of self-control than their less successful peers. This underlines the importance of self-control as an important personality factor for success. These findings might have implications for talent selection and for sport psychological training.

Sporting federations invest a lot of resources in finding talents that might later develop into world class athletes. For example, in their development program which is aimed at 11–14 year old children, the German football federation (Deutscher Fussball Bund; DFB) invests about ten million Euros per year with the stated goal to give every talent “the same chance to be scouted, developed, and sponsored (DFB, 2019b).” However, “it is hard, if not impossible, to identify future top performers at a very early age (Toering and Jordet, 2015, p.1).” The development of youth talent is affected by multiple genetic and environmental influences and inter-individual variation in physical maturation further reduces the predictive validity of talent identification programs (e.g., Vaeyens et al., 2008). Consequently, researchers have emphasized the need to focus more on the development of expertise (Toering and Jordet, 2015). Irrespective of the sporting discipline (or indeed any other domain), expertise development relies on deliberate practice (Ericsson et al., 1993). Indeed, the amount of deliberate practice athletes accumulate is linked to the level of expertise they achieve (Baker and Young, 2014; but see also Macnamara et al., 2016).

Training to become an elite athlete can take more than 20 years (Walsh, 2014), and the road to success might not be a linear one. In addition, dedication to training relies on effort and athletes have to delay more immediate gratifications (e.g., going out with friends) to achieve their long-term goal of becoming an elite athlete (Tedesqui and Young, 2017). Consequently, researchers have become interested in personality traits that might facilitate the accumulation of expertise in sports (e.g., Bartulovic et al., 2017; Tedesqui and Young, 2017). One such trait might be self-control. Self-control refers to the effortful regulation of spontaneous impulses in order to bring behavior in line with valuable goals (Duckworth, 2011). Self-control has been conceptualized as a state and a trait (Tangney et al., 2004). Research on state self-control has repeatedly shown that self-control affects performance in different sporting disciplines (for a review, please see Englert, 2016). For example, impaired performance has been found in sprint-running and time trial cycling if participants had performed a self-control demanding task before (Englert and Wolff, 2015; Englert et al., 2015). Thus, research on state self-control indicates that self-control is needed to perform a single bout of exercise at a high level, which might in the long run help in the attainment of expertise because the quality of deliberate practice might be enhanced. In addition, self-control has consistently been associated with successful goal-striving and the foregoing of immediate impulses in the service of a more important long-term goal in various domains (e.g., de Ridder et al., 2012; Mischel, 2014). For example, in a 32-year longitudinal study, Moffitt et al. (2011) showed that “childhood self-control predicts physical health, substance dependence, personal finances, and criminal offending outcomes (p. 2693).” Since achieving elite status in sport can also be seen as a challenging long-term goal, we assume trait self-control in youth athletes might be an important contributor to success too.

In regard to trait-self-control, research indicates that self-control might help athletes stick with work out regimes (e.g., Bertrams and Englert, 2013), facilitate the accumulation of deliberate practice (Tedesqui and Young, 2017), and in turn facilitate the attainment of greater levels of expertise (Toering and Jordet, 2015; but see also Tedesqui and Young, 2017). Thus, better athletes might be better at controlling themselves. In line with this, Martin et al. (2016) showed that elite cyclists performed better on a computerized stroop task than their non-elite counterparts. The stroop task is among the most frequently reaction-time based behavioral measures of self-control (Wolff et al., 2018) and has displayed convergent validity with self-report measures of self-control (Duckworth and Kern, 2011). Accordingly, in a study that utilized a self-report measure of trait self-control, Toering and Jordet (2015) found a small positive association between sports performance and self-control in a sample of adult Norwegian soccer players. Using a sample that was more heterogenous in regard to age and sporting discipline, Tedesqui and Young (2017) found a more complex pattern of results: the impulse control facet of self-control was linked to more voluntary practice time and athletes high in self-discipline (a second self-control facet that was analyzed by Tedesqui and Young) reported fewer thoughts of quitting their respective sports. However, differences in self-control were not linked to athletes’ skill levels. Thus, while facets of self-control were linked to variables that contribute to attaining expertise, this was not accompanied by a higher athletic level. Taken together, while trait self-control appears to be important in the sporting context, further research is needed to better understand the exact relationship between high trait self-control, and athletic level (Tedesqui and Young, 2017).

For example, it is unclear if potential differences in trait self-control already exist in the youth ranks or whether differences emerge later as part of being an elite performer. Various studies have shown that already in their first decade of life, their levels trait self-control prospectively predict childrens’ academic success (Vitaro et al., 2005; Moffitt et al., 2011; for an overview on the role of self-control in academic achievement, please see Duckworth et al., 2019). This suggests that individual differences in self-control are meaningfully associated with success already at a young age.

## The Present Study

Across several domains, including sport, trait self-control is associated with favorable outcomes (e.g., de Ridder et al., 2012; Englert, 2016). Less is known if trait self-control differentiates between the successful and the less successful and if such differences are already manifest in youth athletes. However, in light of the difficulty of identifying talent (Vaeyens et al., 2008) it is crucial to investigate personality traits that might facilitate expertise development (Toering and Jordet, 2015; Tedesqui and Young, 2017). Here we set out to test this open question by assessing self-reported self-control in a sample of youth football players that have been selected for the German talent development program and a matched control group of football players from the same clubs. As self-control appears to differentiate the successful from the less successful students in school already at a young age, we expect a similar pattern in regard to sports performance. Thus, we tested the hypothesis that selected youth football players score higher on a standardized measure of trait self-control than youth football players from a control group.

## Methods

### Participants and Procedure

Fifty-two youth football players (*n* = 13 female, *M* = 13.42 ± 1.75 years, range 11–17 years) from Southern Germany participated in the study. All players all came from the same Football district (Lake of Konstanz) and trained at one of four clubs within this district. Within their clubs, some players were either selected (*n* = 25) or not selected (*n* = 27) for the German talent development program. The German talent development program aims at identifying a region’s biggest talents and supporting them to maximize their chances of becoming an elite level adult player. To do so, the DFB employs about 1.200 coaches to identify the most promising youth players that train in one of the 25.000 football clubs in Germany. Within this large-scale approach, about 22.000 talented children and adolescents participate in the program annually^1^. To enhance the chance of being selected for the program, the children can train specific selection-relevant drills that are provided by the DFB as technical skills talents should possess to make it to the program (dfb.de). Talents that are selected for the program receive one extra training session per week by a coach that is appointed by the DFB (DFB, 2019b). After they and their caregivers had given written informed consent at the training facility, the youth athletes were provided with a link to an online questionnaire that consisted of a trait measure of self-control, and demographic variables. The questionnaire could then be completed at home by the players. The procedure followed the guidelines laid out in the Declaration of Helsinki.

### Measures

Trait self-control was measured with the German version (Bertrams and Dickhäuser, 2009) of a well-established measure of self-control, the Brief Self-Control Scale (BSC; Tangney et al., 2004). The BSCS consists of 13 items that have to be answered on a 5-point Likert-type scale with answers ranging from “does not apply at all” to “applies completely.” A sample item is for example “I am good at resisting temptation.” High levels on the BSCS reflect higher trait self-control and total ω was = 0.82 in our sample.

Weekly training volume was assessed with one item, referring to the frequency of their weekly club training: “How many club training sessions do you have per week?” Answers could range between one and five times per week. A second question asked for the duration of each training session: “How long does a session last?” Based on typical training durations in this age group, the answering options 60, 75, 90, 105, or 120 min were provided. If none of those options were correct, players could also enter the duration in a text field. To arrive at weekly training load in minutes, frequency and duration were multiplied for each player.

### Statistical Approach

Here, we use Bayesian inference as opposed to more traditional null-hypothesis significance testing (NHST). Recent years have seen an increase in the use of Bayesian inference (van Doorn et al., 2019) and a rising awareness of the limitations that are inherent to NHST (Kruschke et al., 2012). A Bayesian approach has multiple pragmatic advantages (Wagenmakers et al., 2018), such as the possibility to quantify evidence in favor of the null hypothesis (e.g., Dienes, 2014) and to compare statistical models (e.g., Wagenmakers, 2007). In spite of these advantages, Bayesian inference has not yet been adopted by many sport psychology researchers. Following recent calls (e.g., Kruschke et al., 2012) we applied Bayesian statistics. We employed easy to use open-source software (JASP; JASP Team, 2018) and we followed the recommendations laid out by van Doorn et al. (2019).

## Results

### Sample Characteristics

The Bayes factors for comparing the selected (*M*_*a*__*ge*_ selected = 14.20, *SD*_*age*_ selected = 1.71) and non-selected groups (*M*_*age*_ non-selected = 14.63, *SD*_*age*_ non-selected = 1.80) provide no evidence for differences in regard to age (*Bayes factor* = 0.38) or sex (selected = 72% female, non-selected = 77.8% female, *Bayes factor* = 0.33). Expectedly, comparing the weekly training minutes of the selected group (*M*_*training*_ selected = 262.8, *SD*_*training*_ selected = 67.10) and the non-selected group (*M*_*training*_ non-selected = 196.7, *SD*_*training*_ non-selected = 60.05) provided very strong evidence for a higher training load in the selected group (Bayes factor = 59.79).

### Inference Statistics

**FIGURE 1:**
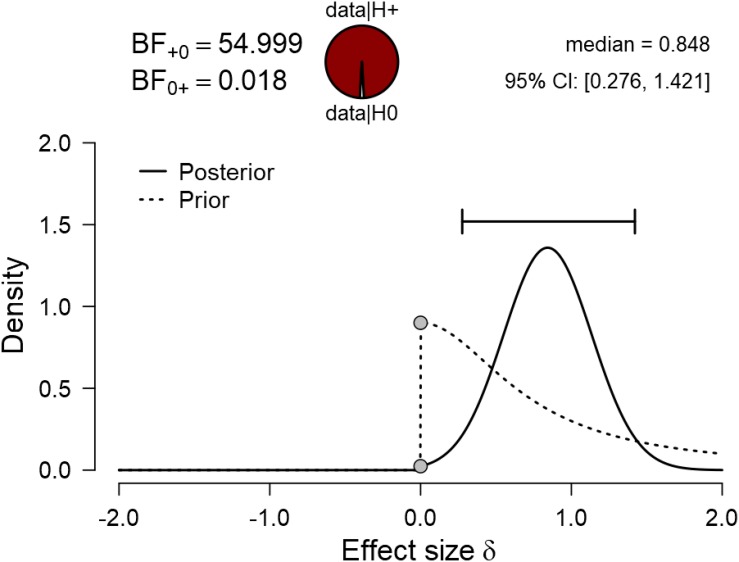
Results of the Bayesian two-sample *t*-test.

**FIGURE 2:**
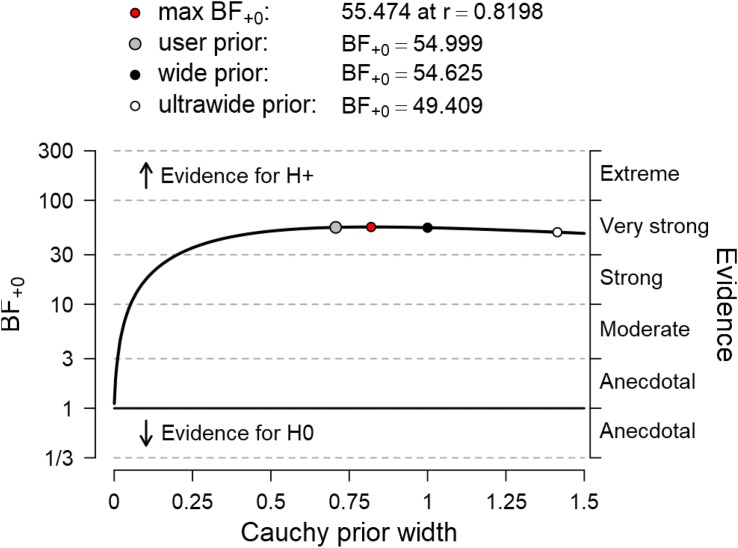
The Bayes factor robustness test shows that the obtained results are relatively robust against different prior specifications.

To test the hypothesis that elite athletes display higher levels of self-control than their non-elite peers, we performed a one-sided Bayesian two-sample *t*-test, and we report the Bayes factor to quantify evidence for this hypothesis (Figure 1). The Bayes factor obtained from this analysis is 54.99, indicating very strong evidence in favor of the hypothesis that elite youth football players indeed have higher trait self-control (*M* = 3.75, *SD* ± 0.47) than non-elite youth football players (*M* = 3.33, *SD* ± 0.40). As an estimate for the effect size and the uncertainty regarding its size, we report the 95% credible interval around the posterior distribution of the standardized effect size δ obtained from this *t*-test. The credible interval shows that, under the assumption that the effect exists, its true size is between 0.28 and 1.42. One frequent critique of Bayesian statistics is the subjectivity that can be introduced by the prior specification. We report the Bayes factor robustness test to assess how the Bayes factor would change if priors of different width had been chosen. As can be seen in Figure 2, the Bayes factor remained relatively stable across various prior specifications, indicating the robustness of our findings.

## Discussion

We found that youth football players that were selected for a talent development program display higher levels of self-control than their non-selected peers. This extends previous research on the importance of self-control in sports by highlighting that self-control differences are evident already at a very young age. Further, our findings add to the emerging pattern that personality differences are important aspects of athletic success (Allen et al., 2013). More specifically, they add to the still relatively scarce body of literature that investigates the role of trait self-control in regard to athletic success (Toering and Jordet, 2015) and expertise development via deliberate practice (Tedesqui and Young, 2017).

One can think of two possible explanations for these findings. On the one hand, the sporting environment, with its emphasis on foregoing immediately gratifying activities (e.g., watching TV) and instead putting effort into activities that might not offer immediate gratification (e.g., going for an endurance run) but that make long-term success more likely, might affect trait self-control. Thus, more talented athletes might be more frequently exposed to self-control demanding situations and develop trait self-control as a consequence of their athletic involvement. According to this view, higher trait self-control is not a prerequisite for achieving elite level but rather a consequence of involvement in such demanding contexts. This is in line with research on the positive effect of childhood sports participation (for children as young as 6 years old) on positive personality development (e.g., Allen et al., 2015). In addition, research on the effectiveness of self-control training provides preliminary evidence that self-control can indeed be improved if people repeatedly apply self-control (for a meta-analysis, see Friese et al., 2017). However, it must be noted that the average effect size of these trainings is relatively small (Friese et al., 2017) and personality tends to be quite resistant to change, getting more stable as a function of age (Anusic and Schimmack, 2016). In line with this, it has been suggested that self-control is quite stable after the age of ten (Hirschi and Gottfredson, 2001; Turner and Piquero, 2002), which renders big improvements in self-control due to sports participation less likely.

On the other hand, due to the self-control demands that are imposed by the sporting environment, youth athletes with inferior self-control might simply be more likely to drop out of talent development programs because they might struggle to consistently invest the effort needed for keeping up with higher demands. In line with this, higher trait self-control is associated with reduced perceptions of mental effort in physically demanding tasks, indicating a more efficient management of self-control demands (Wolff et al., 2019a). This explanation also fits well with the proposition from expertise development that emphasizes the need for effortful deliberate practice (Ericsson et al., 1993) and the finding that better athletes indeed tend to have accumulated more deliberate practice (Baker and Young, 2014). After all, a highly competitive environment like elite sports requires youth athletes to refrain from attractive short-term goals (e.g., sleeping longer in the morning) and instead to invest effort toward their long-term goal (e.g., putting in an early morning training session before school). Thus, those who are better at investing and tolerating effort might simply be better equipped to stay in this environment. This is also in line with longitudinal research in the academic setting showing that self-control is a significant predictor for academic success (e.g., Duckworth et al., 2019) and that self-control is linked to better exercise adherence (e.g., Stork et al., 2017).

### Limitations and Future Research

Taken together, our study indicates that selected and non-selected youth football players differ in regard to trait self-control. However, it has yet to be investigated what this difference means, as our study was not designed to test the validity of either of the explanations described above. Further, both explanations must not be mutually exclusive: While it appears likely that self-control helps youth athletes to reach elite status, it cannot be ruled out that the sporting environment further strengthens trait self-control. To unravel how self-control and the development of elite athletic level are related, cross-sectional studies do not suffice. Since experimental studies are not suitable for the investigation of this relationship, only longitudinal designs remain, which are carried out with sufficiently large samples of elite youth athletes over extended periods of time, so that sophisticated statistical methods can be applied (Duckworth et al., 2010). The present finding can be understood as an indication that such extensive and costly research may be worthwhile. Such future research, which also addresses the underlying processes, could help to further optimize the promotion, selection, and training of elite athletes.

Second, although research points toward trait self-control as being an important individual difference variable for athletic success (Toering and Jordet, 2015; Martin et al., 2016), this has not been found in all published studies. For example, although they found a relationship between trait self-control and deliberate practice variables, Tedesqui and Young (2017) did not find differences in self-control variables between more or less skilled athletes. While it is difficult to make comparisons across studies, two differences between the present study and the study by Tedesqui and Young (2017) are obvious and might provide an impetus for further research. First, the sample of Tedesqui and Young was composed of athletes who were from different sports and primarily from individual sports (68.8%) as opposed to our football-only sample. Future research might directly focus on assessing differences between individual and team sports or differences between skill-based and more endurance determined sports to understand if the role of trait self-control varies between sports. Second, other researchers have utilized the BSCS to capture two factors of self-control (Toering and Jordet, 2015; Tedesqui and Young, 2017), whereas we chose to use the one-factorial solution. While there is research suggesting that the BSCS indeed captures two distinct facets of self-control (e.g., Maloney et al., 2012), a study on the factorial structure of the German variant did not find support for this two-factorial solution and the authors recommended using the unimodal score instead (Lindner et al., 2015). Thus, the potential dimensionality of trait self-control and its associated measures should also be a target for future research to establish if possible facets of trait self-control are differentially related with athletic success. In addition, researchers have focused on the positive effects of self-control (e.g., Moffitt et al., 2011) and generally high-self-control is held in high regard (Duckworth, 2011). However, since the exertion of self-control induces fatigue and feelings of frustration (Wolff et al., 2019b), one cannot rule out that the need to chronically employ self-control to stay in a highly competitive system like elite sports might negatively affect motivational and emotional states of youth athletes.

Taken together, while the initial evidence regarding the importance of trait self-control for athletic success is promising, it is clear that further research is needed to better understand the specific role of self-control on sporting excellence.

## Data Availability Statement

The datasets generated during and/or analyzed during the current study are available from the corresponding author on reasonable request.

## Ethics Statement

The procedure followed the guidelines laid out in the Declaration of Helsinki (1975). Participation was voluntary and happend only after participants and their caregivers had given prior written informed consent. The present study falls outside the range of research requiring an IRB statement according to the position of the ethics committe of the University of Konstanz which states (https://www.uni-konstanz.de/en/university/administration-and-organisation/university-bodies-and-committees/university-bodies-for-scientific-integrity/ethics-committee/): “The Ethics Committee does not address questions (…), which do not affect the health, dignity or personal rights of human test subjects.” This is the case for the present study as participants were not subjected to any experimental manipulation and only completed an anonymous questionnaire.

## Author Contributions

WW conceived the study design and discussed it with AB and JS, conducted the study and performed the statistical analyses, and wrote the first draft of the manuscript. AB and JS revised and finalized the manuscript.

## Conflict of Interest

The authors declare that the research was conducted in the absence of any commercial or financial relationships that could be construed as a potential conflict of interest.

## Footnotes

1 For reference, in 2019, more than 1.8 million boys and more than 300.000 girls were listed as DFB members (DFB, 2019a).
